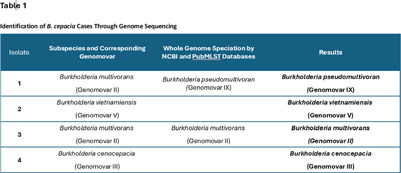# Importance of a Comprehensive Infection Prevention Approach During an Outbreak Investigation of Burkholderia cepacia

**DOI:** 10.1017/ash.2025.374

**Published:** 2025-09-24

**Authors:** S. Elizabeth Alvarez, Kay Sams, Leslie Montalvo Gonzalez, Jose Alexander

**Affiliations:** 1 Adventhealth

## Abstract

**Background:** Burkholderia cepacia (B. cepacia) is an aerobic, gram-negative bacilli commonly found in soil and water that has been linked to healthcare-related outbreaks. Between April 25 and September 6, 2024, four cases of B. cepacia were investigated at an acute tertiary healthcare facility. Three of the patients had undergone inpatient cardiac procedures, and the fourth patient had a tracheostomy procedure. All culture sources were collected from the respiratory tract and revealed the presence of B. cepacia. A 12-month hospital surveillance retrospective review had not previously identified this organism in our inpatient population. This investigation utilized a comprehensive approach to identify potential modes of transmission. **Method:** A multidisciplinary team comprised of nursing, respiratory therapy, anesthesia, and infection prevention was formed to enhance communication and identify potential gaps in infection prevention processes that may have contributed to recent cases. We concentrated on identifying potential reservoirs related to respiratory practice, with a specific focus on the use of ventilator equipment and supplies which included researching the manufacturer instructions for use (MIFU). Our investigation also included observations in the operating room and Intensive Care Unit (ICU), adenosine triphosphate (ATP) sampling of environmental surfaces, implementation of empiric contact isolation precautions in the ICU, ensuring access to alcohol-based hand sanitizer, and personal protective equipment (PPE). The four respiratory samples of B. cepacia were sent to an outside laboratory for genotyping. **Results:** High ATP readings in the operating rooms indicated a need for additional environmental cleaning. Issues were identified with improper use and storage of respiratory equipment which included transport ventilators, patient supplies, and anesthesia equipment. Incorrect use of PPE by perioperative and ICU staff was also observed. Genome analysis of the samples confirmed no microbiological correlation or epidemiological link (see Table 1). **Conclusion:** Although results indicate that internal transmission was unlikely, this investigation highlights the importance of conducting a comprehensive outbreak investigation to identify potential gaps in processes that could contribute to modes of transmission. This understanding allowed the implementation of targeted interventions to address the issues and prevent future cases.

**Figure:**